# Mechanical and Microstructural Characterization of Trapezoidal Corrugated-Core Al Sandwich Panels Under Quasi-Static Compression

**DOI:** 10.3390/ma19030548

**Published:** 2026-01-30

**Authors:** Alessandra Ceci, Girolamo Costanza, Maria Elisa Tata

**Affiliations:** Department of Industrial Engineering, University of Rome Tor Vergata, 00133 Rome, Italy; alessandra.ceci@uniroma2.it (A.C.); elisa.tata@uniroma2.it (M.E.T.)

**Keywords:** sandwich panels, trapezoidal/corrugated core, aluminum 3000 series, quasi-static compression, energy absorption, microstructure, SEM/EDS, XRD

## Abstract

Sandwich panels with trapezoidal (corrugated) cores combine low weight, high specific stiffness, and energy absorption capability. This study analyzes four configurations with different core heights by means of microstructural analyses (optical microscopy, SEM/EDS, XRD) and quasi-static compression tests. The tests yield stress–strain curves with an initial linear stage, a peak, a plateau, and a densification stage. Peak stresses range from 0.5 MPa for the thickest core (P1) to 6.2 MPa for the thinnest core (P4), while the energy absorbed density (EAD) increases with strain: at ε = 30% it varies from 0.031 to 0.670 J/cm^3^, and at ε = 50% the thin-core configuration reaches ≈1.113 J/cm^3^. The face sheets and the core are both manufactured from AA 3000 series (Al–Mn) aluminum alloy; widespread micro-porosity and Fe/Mn-rich phases are observed by SEM/EDS. XRD confirms aluminum with different peak intensities ascribable to the manufacturing texture. Increasing the core height promotes earlier local/global instabilities and reduces the peak stress; the thinnest core displays higher stiffness and peak loads. These findings support the use of trapezoidal corrugation where low weight and progressive strain are required.

## 1. Introduction

Sandwich structures are a fundamental class of engineered composite materials that are particularly valued in applications requiring a high stiffness-to-weight ratio and excellent structural strength [1,2]. These structures are recognized as lightweight, multifunctional components, typically consisting of two thin, stiff outer face sheets separated by a low-density core [3]. Their broad range of applications includes critical sectors such as aerospace, marine, civil construction, and automotive, where, for example, hierarchical structure improves heat transfer efficiency [4].

Among the various cellular core configurations, such as honeycomb or foam, corrugated cores emerge as the preferred lightweight materials. Their geometry provides highly directional mechanical properties and remarkable crush resistance, making them ideal candidates for impact/blast load mitigation [5,6]. The geometric forms of such cores vary and include sinusoidal, triangular, rectangular, and, of particular interest in the present study, trapezoidal shapes [7,8]. The specific corrugated-core configuration directly influences the ultimate strength, failure modes, and energy absorbed density (EAD) of the panel [6]. It has been shown that trapezoidal-core structures generally offer higher strengths in compression and shear tests [9].

Sandwich panels with trapezoidal corrugated cores have been extensively investigated, both experimentally and numerically, to fully assess their potential under complex loading scenarios. A significant part of the research has focused on low-velocity impact response. Kılıçaslan et al. [5] examined the behavior of aluminum layered panels (with trapezoidal corrugated cores and intermediate aluminum foil layers) using spherical, flat, and conical impactors and validated experimental results with finite element (FEA) simulations in LS-DYNA. They observed that, for the same impact velocity, the panels impacted with spherical and flat indenters were not perforated and exhibited slightly higher energy absorption and deformation forces in the 0°/90° corrugation orientation compared with 0°/0°. It was also noted that structures with lower-strength cores, e.g., brazed panels compared to bonded ones, tended to spread deformation over a larger area.

In parallel, research has been extended to air blast loading. P. Zhang et al. [10] experimentally investigated the influence of key geometric parameters, such as stand-off distance (SoD), face thickness, and corrugation angle, on the deformation and failure mechanisms of laser-welded metallic trapezoidal-core panels. The results showed that reducing stand-off distance increases deflection and damage. Increasing the core web thickness and corrugation angle proved effective for improving blast resistance, reducing the deformation.

A novel production method under vacuum brazing was proposed by Zhang et al. [11] in order to obtain superior properties of the competing core topologies with superior compressive strength in the high-density region.

Under quasi-static loading, attention has been focused on longitudinal bending. Xia et al. [12] compared the performance of different corrugated-core shapes (including trapezoidal) under three-point bending, observing two main deformation modes (Mode I and Mode II) depending on the wall thickness. The trapezoidal panel was found to be efficient and was used for targeted optimizations aimed at maximizing energy absorbed density and minimizing intrusion depth. In the context of bio-based materials aligned with circular economy (CE) principles, Choupani Chaydarreh et al. [6] fabricated trapezoidal-core panels using oil camellia nut shells mixed with poplar particles or fibers, finding that particle-based panels (PBPs) exhibited superior flatwise compression strength and EAD compared with fiber-based panels (FBPs), indicating that particles provided a stronger slope to the trapezoidal structure.

To reliably analyze structural performance, particularly in the regime of large deflections and geometric nonlinearity, advanced numerical methodologies have been developed. Peng et al. [13] presented a nonlinear analysis of sandwich plates with corrugated cores (trapezoidal and sinusoidal) using the Element-Free Galerkin Method (EFGM). This meshless approach, based on first-order shear deformation theory (FSDT) and von Kármán theory, was specifically developed to overcome the mesh distortion issues typical of the Finite Element Method (FEM) in nonlinear, large-deflection problems. EFGM was shown to achieve satisfactory accuracy with a significantly reduced nodal discretization (507 nodes) compared with ANSYS simulations, which required thousands of nodes.

Complementing direct methods, homogenization remains crucial for modeling sandwich structures at larger scales, although it is well known to be more effective in capturing global effects (average deformations) than local effects (such as stresses near welds or concentrated loads) [14,15]. Garbowski & Gajewski [16] improved the homogenization methods by extending strain-energy equivalence between the detailed 3D model and a Reissner–Mindlin plate representation to robustly compute not only extensional and bending stiffnesses but also transverse shear stiffnesses, which are essential for an accurate equivalent model.

Beyond structural strength, trapezoidal cores have been explored for their multifunctional properties. In acoustics, recent studies conducted a vibro-acoustic optimization of panels with corrugated cores (with trapezoidal cavities) to maximize Sound Transmission Loss (STL). They employed the wave-based method (WBM), which proved significantly more computationally efficient than FEM (reducing CPU time by up to 80% at equal accuracy) and thus well suited to optimization procedures requiring heavy computation.

To further enhance mechanical performance, particularly energy absorption and stability, hybrid structures have been developed. Aluminum foam filling of corrugated-core voids has been proposed as a solution to prevent core web buckling [17]. The foam provides lateral support, which reduces the buckling wavelength, inducing a complex coupling effect and altering the deformation mechanism from a single folding to multiple plastic hinges, leading to a significant increase in specific stiffness and energy absorption.

Finally, fabrication techniques are evolving. Fully metallic structures with complex corrugated cores (e.g., V- or X-shaped geometries) are now produced via Wire Arc–Directed Energy Deposition (WA-DED), an additive manufacturing (AM) technique that enables integrated fabrication of lightweight alloys such as Mg VW63K alloy [18]. Moreover, in line with the growing interest in sustainability, new studies have explored the feasibility of producing trapezoidal-core panels using agricultural and forestry by-products, such as crop residues and oil camellia shells [19,20].

In summary, the trapezoidal corrugated core is a versatile configuration that has demonstrated outstanding performance under static and dynamic loads. However, although understanding of its behavior has advanced thanks to complex modeling techniques (such as EFGM and WBM) and geometric optimization [3], the engineering challenge continues to lie in harmonizing multiple objectives (e.g., maximizing EAD and minimizing weight) and in the predictive accuracy of models in the presence of local and nonlinear effects. The main goal of this work is the analysis of the mechanical behavior in static compression of sandwich panels with different geometries, in particular correlating the trapezoidal-core structure with the deformation way, stress–strain behavior, and energy absorbed density.

## 2. Materials and Methods

Four different aluminum sandwich panels were tested, each characterized by a distinct trapezoidal-core height. All panels consisted of two thin outer face sheets and an internal core composed of trapezoidal cavities aligned with the direction of compression. AA 3003 H16 aluminum alloy was employed for both sheets and core. The corrugated core was created by slicing the corrugated monolithic core and positioning it on the panel’s surface plate in various directions and angles. Trapezoidal geometry was chosen as the core form. The geometrical parameters of the core unit cells are given in Table 1. The metal sheets were cut in their final arrangement. The sandwich panel corrugated cores were then fabricated from cut aluminum sheets using a custom-built bending mold. Each sandwich panel sample was created by repeating several unit cells in the transverse and longitudinal directions. The core and surface plates were joined with Araldite 2015 two-component epoxy adhesive, a common bicomponent and thixotropic structural glue room-temperature curing. To verify that the adhesive was thoroughly cured, the prepared samples were maintained at room temperature for 24 h with a predetermined press force applied.

**Figure 1:**
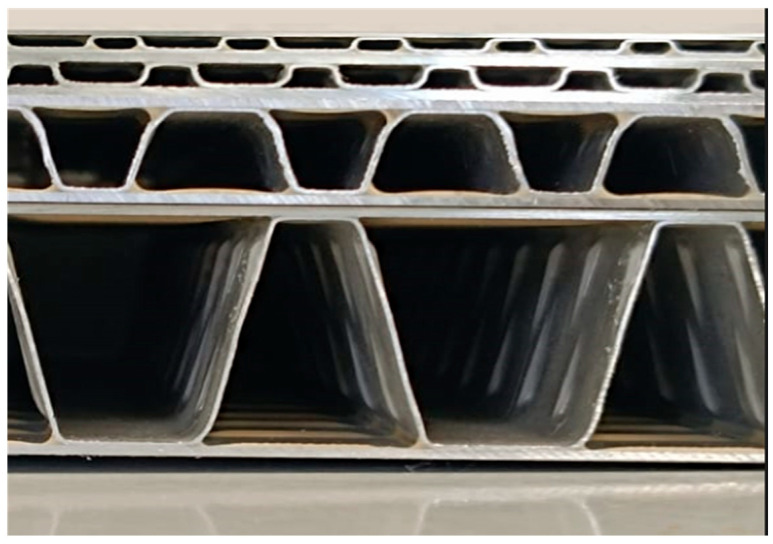
Lateral view of the four sandwich panels tested, from 1 (bottom) to 4 (top).

**Figure 2:**
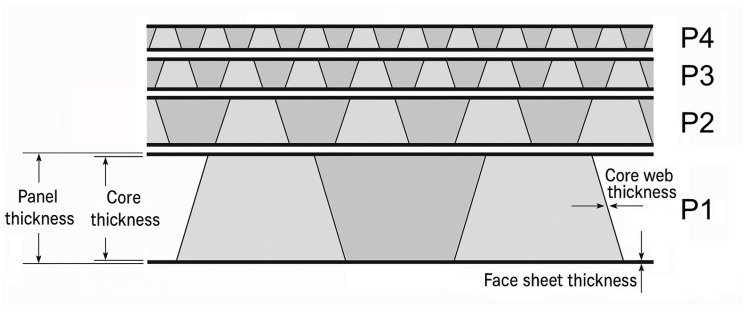
Schematic representation of the sandwich panels with trapezoidal core, showing the main geometric parameters used for characterization: core thickness (h), face sheet thickness (t) and the top and bottom widths of the trapezoidal cavities.

Figure 1 shows a lateral view of the specimens, labeled from 1 to 4 (bottom to top), arranged in descending order of core height. The in-plane dimensions of the samples range approximately from 82 × 84 mm^2^ to 93 × 92 mm^2^, as reported in Table 1. A sketch of the cross-section view of the corrugated board sandwich panel is reported in Figure 2.

### Skin and Core

The skin and core of the corrugated sandwich panels were characterized by means of optical microscope and SEM-EDS analysis. Compression tests were performed by means of an MTS Insight universal machine (maximum load 50 kN) according to the ASTM C365/C365M-22 standard [21].

X-ray diffraction analysis was carried out on skin and core flat samples employing Mo-kα radiation (wavelength 0.071 nm) on a θ–2θ diffractometer with counting steps 0.05° (2θ) and counting time 10 s.

## 3. Microstructural Characterization

**Figure 3:**
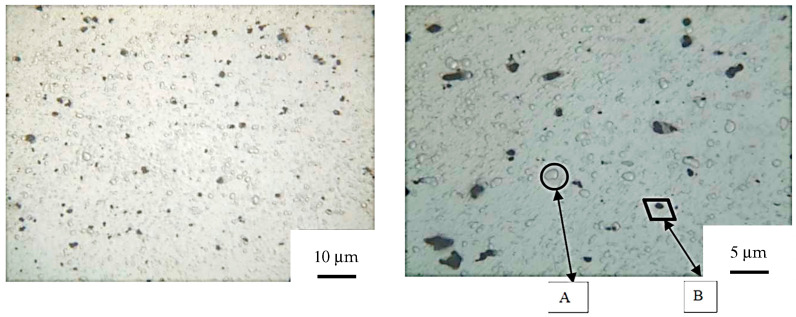
Optical micrographs of the aluminum alloy skins used in the sandwich panels, taken at different magnifications (left 500×, right 1000×). (**A**) Fe-rich and (**B**) Mn-rich particles.

The optical micrographs (Figure 3), at two magnifications, reveal a relatively uniform microcrystalline structure with a distributed small amount of porosity and a large amount of second phases. These features are consistent with typical microstructures of 3000 series aluminum alloys (1.5% Mn, 0.2 Si, to balance Al) manufactured by rolling.

**Figure 4:**
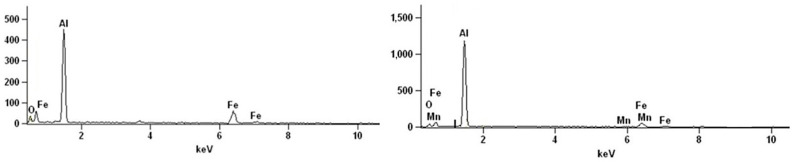
Energy-Dispersive X-ray Spectroscopy (EDS) on point A (**left**) and surface B (**right**) of Figure 3.

To complement the morphological analysis, a qualitative chemical assessment was performed using Energy-Dispersive X-ray Spectroscopy (EDS) (SEM Phenom XL G2, Alfatest, Eindhoven, The Netherlands). Figure 4 shows an EDS spectrum corresponding to points A and B of the micrographs in Figure 3. Aluminum is clearly the dominant element, accompanied by iron (Fe) in the bright particles (A), corresponding to Fe-rich intermetallic phases, and by manganese (Mn) in the particles (B), corresponding to Mn-rich phases (Mn-Al6), in agreement with literature data [20].

**Figure 5:**
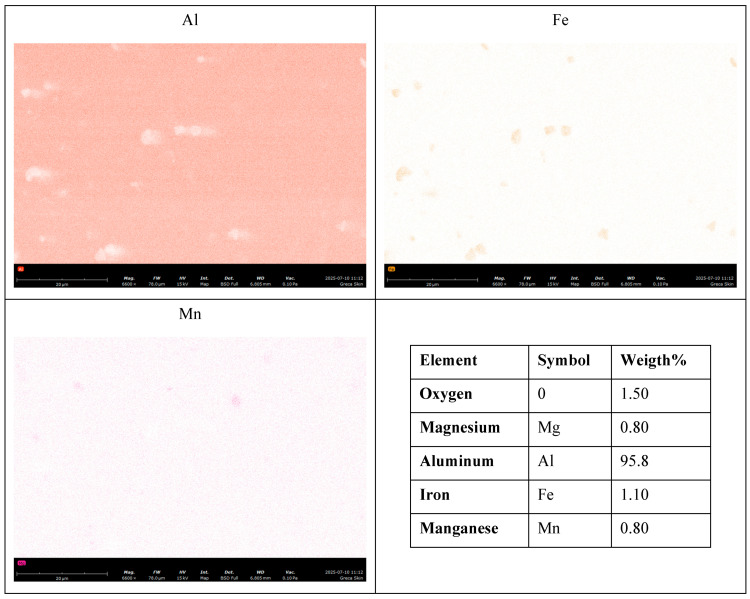
Energy-Dispersive X-ray Spectroscopy (EDS) maps of Al, Fe and Mn respectively, acquired at 5000× magnification. The lower right panel of the figure shows the average composition, by Energy-Dispersive X-ray Spectroscopy (EDS), of the same area of the three maps of Al, Fe and Mn.

The composition of the matrix, reported in the lower right panel of Figure 5, is mainly composed of aluminum (Al), magnesium (Mg), iron (Fe), oxygen (O), and manganese (Mn) and consistent with a commercial aluminum alloy 3000 series, commonly used for structural components requiring moderate mechanical strength and good formability, potentially produced through industrial-scale rolling or extrusion. Based on literature data [20], it is possible to identify the material as belonging to the 3000 series aluminum alloys, such as EN AW-3003 (Al–Mn), commonly used in lightweight structural and automotive components. The oxygen likely originates from surface oxide layers. Magnesium, manganese, and iron may be present as trace elements or mechanical property enhancers.

The combined optical and SEM analyses reveal a relatively homogeneous microstructure, with widespread presence of micro-porosities and intermetallic phases.

The chemical composition of the sample was assessed using Energy-Dispersive X-ray Spectroscopy (EDS). The EDS analysis (bottom right panel of Figure 5) of the sandwich panel skins revealed a composition mainly of aluminum (Al), magnesium (Mg), iron (Fe), oxygen (O), and manganese (Mn).

**Figure 6:**
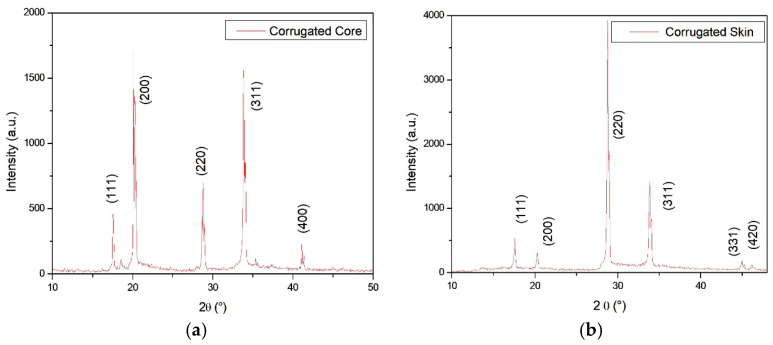
XRD spectra collected on the corrugated core (**a**) and the skin (**b**).

The XRD patterns illustrated in Figure 6a,b, both for skin and core, show peaks corresponding to those of the Al fcc phase. By comparing relative peak intensities, it is evident that the core shows 311, 200 and 220 preferential orientations while the skin exhibits 220 and 311 preferential orientations.

## 4. Compression Tests

**Figure 7:**
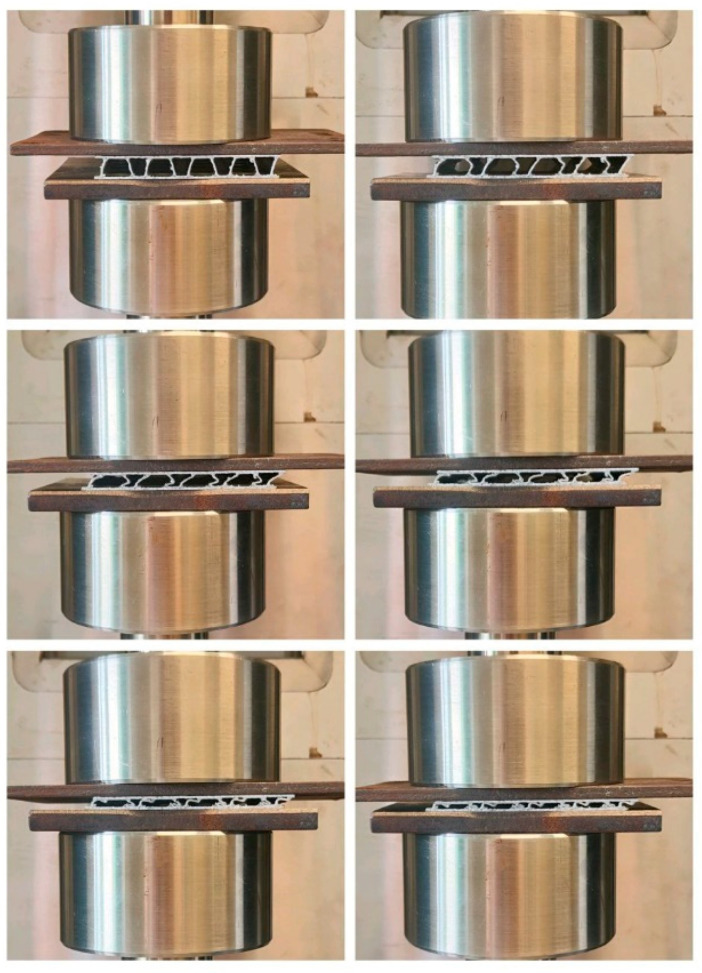
Sequence of compression steps for the sample P2 (10 mm height).

Compression tests were carried out with an MTS Insight universal testing machine with a maximum load capacity of 50 kN calibrated according to ASTM E4-24 [22] (equivalent to Class 1 ISO 7500-1:2018 [23]). The tests were performed under crosshead-control mode, applying a constant crosshead speed of 5 mm/min. The samples were compressed between two steel plates, ensuring a uniform load distribution on the top and the bottom surfaces. Figure 7 shows a typical deformation sequence captured during the compression.

For each geometric configuration (defined by the thickness of the outer face sheets), three specimens were tested to assess the repeatability of the results. Stress–strain curves were derived from the force–displacement data, normalized by the cross-sectional area and initial specimen height.

The following parameters were considered in the analysis:

- Initial stiffness, defined as the slope of the elastic region;
- Maximum stress, corresponding to the peak load before load drop;
- Plateau region (if present), associated with progressive strain;
- Densification, indicated by a sharp stress increase at high strain.

During the tests, force and displacement were continuously monitored and recorded. The acquired load–displacement data were converted into engineering stress–strain curves by considering the external sectional area of the sandwich panels (panel area values, *A_ext_*, reported in Table 1). The engineering stress σ and strain ε were calculated as in Equations (1) and (2):

(1) $$\sigma =\frac{F}{A_{ext}}$$

(2) $$\varepsilon =\frac{∆h}{h_{0}}$$

$h_{0}$ denotes the initial thickness of the sandwich specimen measured along the loading direction prior to the compression test. This value corresponds to the overall height of the sandwich panel, including both face sheets and the trapezoidal core. The term Δh represents the axial displacement during the test, measured as the reduction in specimen thickness under compressive loading. It is defined as the difference between the initial thickness $h_{0}$ and the instantaneous thickness at a given loading stage.

The energy absorption capability of the sandwich panels was evaluated from the engineering stress–strain response obtained under quasi-static compression. The energy absorbed up to a prescribed maximum strain $\varepsilon_{\max}$ was calculated as the area under the stress–strain curve, as in Equation (3).

(3) $$\begin{array}{cccc} & W(\varepsilon_{max})=\int_{0}^{\varepsilon_{max}}\sigma (\varepsilon )\;d\varepsilon & & \end{array}$$

To enable comparison between sandwich configurations with different geometrical characteristics, the absorbed energy was normalized by the external volume of the specimen, leading to the definition of the volumetric energy absorption (EAD), as set out in Equation (4):

(4) $$\begin{array}{cccc} & EAD\left(\varepsilon_{max}\right)=\frac{W\left(\varepsilon_{max}\right)}{V_{\mathrm{ext}}}, & & \end{array}$$

where $V_{\mathrm{ext}}=A_{ext}\cdot h_{0}$ is the external volume of the sandwich panel. The EAD, expressed in J/cm^3^, represents a direct measure of the energy dissipation capability per unit occupied volume.

## 5. Experimental Results

**Figure 8:**
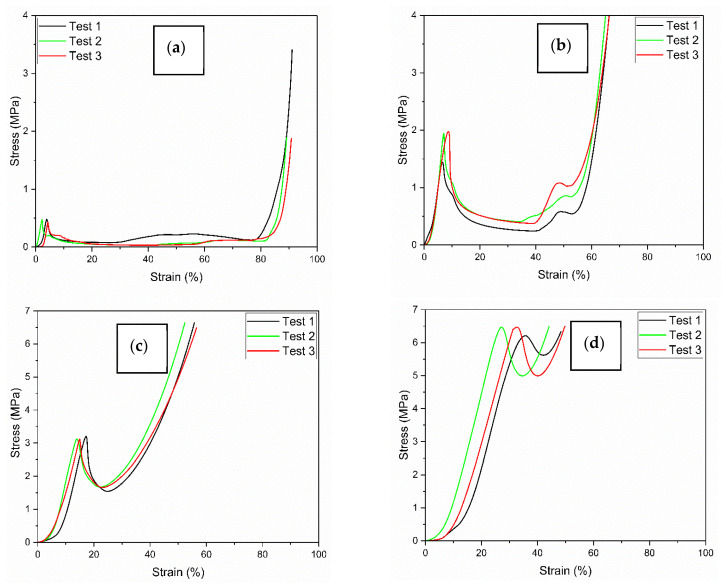
Stress–strain curves (**a**) P1, (**b**) P2, (**c**) P3, (**d**) P4. Each graph shows the results of three repeated tests (Test 1, Test 2, Test 3).

Table 2 summarizes the main mechanical properties obtained from the stress–strain curves (Figure 8) for the four sandwich panel configurations with increasing core thickness. A clear trend of increasing peak stress is observed as the core thickness decreases, ranging from approximately 0.5 MPa for sample P1 (thickest core) to about 6.2 MPa for sample P4 (thinnest core). This trend is consistent with the greater load-bearing capacity provided by the thicker core geometry.

The strain at peak also slightly increases, suggesting a moderate increment in ductility and energy absorption before load drop. The estimated initial stiffness (approximated from the slope of the linear elastic region) also decreases with thickness, indicating a stiffer initial response for thinner panels. Overall, these results confirm that increasing the core thickness significantly affects the mechanical properties of the sandwich structure.

The values reported in Table 2 are averaged over n = 3 tests per sample. The estimated standard deviations are as follows:

- Elastic strain, ε_el_ (%): P1 ≈ 0.3; P2 ≈ 0.6; P3 ≈ 0.5; P4 ≈ 0.8;
- Elastic stress, σ_el_ (MPa): P1 ≈ 0.03; P2 ≈ 0.07; P3 ≈ 0.10; P4 ≈ 0.15;
- Strain at peak, ε_peak_ (%): P1 ≈ 0.3; P2 ≈ 0.5; P3 ≈ 0.7; P4 ≈ 1.8;
- Peak stress, σ_peak_ (MPa): P1 ≈ 0.03; P2 ≈ 0.08; P3 ≈ 0.12; P4 ≈ 0.35;
- Estimated stiffness (MPa): P1 ≈ 0.6; P2 ≈ 0.7; P3 ≈ 0.8; P4 ≈ 1.0;
- Plateau stress, σ_plateau_ (MPa): P1 ≈ 0.02; P2 ≈ 0.03 (not applicable to P3–P4).

For each metric, the coefficient of variation (CV = SD/mean) is reported too. CVs are generally low to moderate: for elastic strain, CV ranges from 3.3% to 8.6%; for elastic stress, from 3.8% to 7.5%; for strain at peak, from 4.0% to 7.7%; for peak stress, from 3.8% to 6.0%; and for estimated stiffness, from 4.6% to 5.4%. Plateau stress shows CVs of 9.5% (P1) and 6.7% (P2). These values indicate limited scatter for most quantities and slightly higher variability in the plateau region.

**Figure 9:**
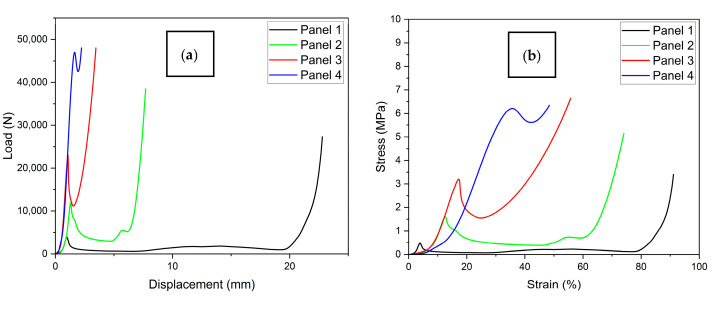
Experimental results from quasi-static compression tests on corrugated-core aluminum sandwich specimens. (**a**) Load–displacement curves, (**b**) Stress–strain curves.

Figure 9 presents the results of the quasi-static compression tests carried out on the corrugated-core sandwich panels. In particular, Figure 9a reports the load–displacement curves, while Figure 9b shows the corresponding stress–strain curves up to large deformation levels. The mechanical response is characterized by an initial linear elastic region, followed by a nonlinear regime with stress fluctuations and, in some cases, strain localization phenomena. The curves show variability among the samples, which is consistent with the inherent manufacturing imperfections typical of open cellular structures. Also, the absence of a well-defined plateau in the stress–strain curves for samples P3 and P4 can be ascribed to a different deformation mode due to the buckling of the internal walls inside the core and an abrupt deformation.

From the stress–strain curves, it can be observed that samples P3 and P4 (correctly identified after correction) exhibited the highest peak stresses and most pronounced hardening behavior, indicating a more progressive collapse mechanism and enhanced energy absorption. Meanwhile, they showed a limited strain (%). On the other hand, sample P1 showed a softer response, with a plateau-like behavior starting from lower stress levels, which may be ascribable to premature local buckling or the early onset of plastic deformation. The same considerations, but less pronounced, apply to sample P2.

The energy absorption density (EAD), reported in Table 3, is defined as the energy absorbed by the structure during deformation, normalized by its volume. It is a key indicator of the impact resistance and energy dissipation capabilities of cellular or porous structures, especially in applications such as crash absorbers, packaging, or protective equipment. EAD was calculated as the area under the stress–strain curve up to predefined strain levels and expressed in (J/cm^3^). Table 4 reports the measured values for each specimen at 30% and 50% of strain.

The analysis of energy absorbed density (EAD) values reveals a clear and consistent trend: panels with thinner cores (P3 and P4) exhibit greater efficiency in terms of energy absorbed per unit volume. This behavior is particularly pronounced at higher strain levels (50%), where panels P3 reach EAD values exceeding 1 J/cm^3^, while the thickest panel (P1) remains below 0.07 J/cm^3^. The higher standard deviation for EAD found in sample P4 is ascribable to the great variability in the deformation mode and associated buckling of the internal walls.

This trend suggests that, despite a lower overall deformability, more compact structures promote localized collapse mechanisms and effective mechanical energy dissipation. However, the increased variability in results at higher strain levels (i.e., higher standard deviation) indicates that collapse modes become more sensitive to local imperfections and less repeatable. This observation is consistent with the behavior observed in the stress–strain curves and the failure mechanisms discussed in the previous section.

## 6. Correlation of Geometry–Mechanical Properties

To investigate the influence of core height on the mechanical performance of sandwich panels, a theoretical analysis was conducted based on a log–log regression between the core height h and three key mechanical parameters extracted from experimental tests: peak load Fmax, stiffness k, and energy absorption density (EAD).

In classical cellular material theory, such as the model proposed by Gibson [24], several mechanical properties Y scale with the relative density ρ∗/ρs.

However, in the present study, the relative density cannot be directly derived from the core height alone. The four commercial panels investigated differ not only in core height but also in corrugation pitch and trapezoidal cell geometry. As a consequence, core height cannot be interpreted as a direct measure of relative density. Nevertheless, reducing the core height in this family of panels is intrinsically associated with a densification of the corrugation pattern, characterized by a reduced pitch and more compact trapezoidal cavities. This geometric densification increases the effective amount of material per unit area and strongly affects the load-bearing mechanisms of the core. For this reason, core height can be regarded as a meaningful geometric indicator that is strongly correlated with the effective structural compactness and stability of the corrugated structure, even if it does not uniquely define the relative density. Based on this rationale, a log–log regression of the form (Equation (5)),

(5) log(Y)=log(a)+blog(h),

was applied to highlight empirical scaling trends between core height and mechanical response.

This regression is not intended to represent a fundamental density-based scaling law but rather to provide a comparative description of geometric effects within the investigated family of panels.

**Figure 10:**
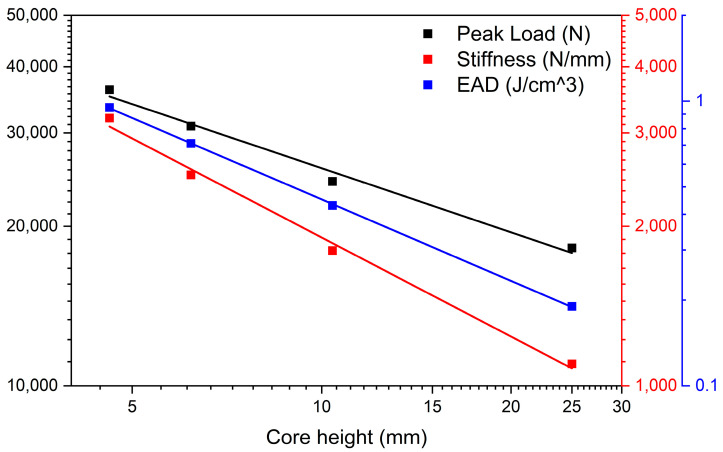
Log–log regression between core height and mechanical properties: peak load (black), stiffness (red) and EAD (blue).

The results of the regression, shown in Figure 10, reveal the following trends (Equation (6)):

Peak load:

(6) $$F_{max}\propto \;h^{-0.16},\;R^{2}=0.95$$

A slight decrease is observed with increasing core height, which can be attributed to enhanced local buckling phenomena in the thickest cores, consistent with previous studies [5,6].

Stiffness (Equation (7)):

(7) $$k\propto \;h^{-0.28},\;R^{2}=0.99$$

This behavior is consistent with the bending-dominated response of sandwich cores: thicker cores tend to reduce global panel stiffness, as described in [24,25].

Energy absorption density (EAD):

$$EAD\propto \;h^{-0.74},\;R^{2}=0.98$$

A significant drop in EAD is observed as *h* increases, indicating that thinner panels are more efficient in terms of energy absorbed density. This trend is in line with observations reported for corrugated and lattice-core panels [26,27,28].

These findings are in good agreement with the actual geometry of the core, shown in Figure 1: the thinner cores (P3–P4), which are the densest, are also those with the highest stiffness, peak stress, and energy absorption. Conversely, the thickest core (P1), with the lowest densification, exhibits the weakest compressive response.

Overall, these results confirm that the trade-off between mass and mechanical performance plays a crucial role in the design of sandwich structures. Thinner configurations offer a favorable compromise for applications where lightweight and energy efficiency are critical, such as in transport, protective structures, and aerospace components.

## 7. Discussion

The stress–strain curves clearly show a strong influence of the core height on the mechanical response of the corrugated sandwich panels. As the core height increases (sample P1), the structure exhibits greater deformability but lower peak stress. In contrast, panels with thinner cores (samples P3 and P4) exhibit higher stiffness and significantly higher peak loads but reduced strain.

Although initially counterintuitive, this trend can be explained by the increased susceptibility of thicker cores to local and global buckling, which reduces their load-bearing capacity. Conversely, in panels with thinner cores, the failure occurs by localized crushing, allowing higher loads before densification.

The initial stiffness, estimated from the slope of the elastic region of the stress–strain curve, confirms that thinner-core panels are stiffer. This is consistent with the more compact structure and the more effective support provided by the tightly packed corrugations.

The energy absorption density values confirm the greater energy absorption capability of thinner-core panels, especially at low strain levels.

Experimental images show distinct collapse mechanisms: in thicker-core specimens, progressive local buckling and gradual crushing dominate, whereas in thinner cores, failure is more abrupt, with localized damage and brittle-like fractures. These behaviors align with the increased stiffness and reduced ductility of thinner-core panels.

The results obtained are consistent with previous findings reported in the literature for corrugated or lattice-core sandwich structures. Several authors have observed that increasing the core height leads to a reduction in global stiffness and a greater susceptibility to local buckling. For example, refs. [10,12] demonstrated that sandwich panels with higher corrugated cores tend to fail prematurely, despite offering greater deformability. Similarly, in the present study, the specimen with the thickest core height (specimen P1) exhibited a significantly lower peak load compared to the one with the smallest height (specimen P4), confirming that increasing height can introduce instability and reduce overall load-bearing capacity.

Moreover, although the energy absorption density increases with strain, the configuration with an intermediate core height demonstrated a good compromise among stiffness, load-bearing capacity, and energy absorption. These findings are consistent with those reported by [29,30,31], highlighting the potential of this type of core for applications where a light weight and energy dissipation are required, such as in transportation and impact protection.

EDS and SEM analyses confirmed the predominance of aluminum with minor traces of Fe, Mg, Mn, and O. The microstructure appears mostly homogeneous, without significant changes between samples that could account for the compressive behavior. This supports the conclusion that the observed behavior is primarily governed by core geometry rather than microstructural differences.

## 8. Conclusions

This study investigated the compressive behavior of sandwich panels featuring a trapezoidal corrugated aluminum core manufactured from a 3000-series alloy and characterized by varying core heights. The experimental results highlighted a strong influence of core height on the mechanical response:

- The peak stress decreases with increasing core height ranging from 6.2 MPa (4.6 mm) to 0.5 MPa (25 mm), indicating the dominance of local buckling phenomena over material strength.
- The global stiffness is reduced in thicker cores ranging from 18.6 MPa (4.6 mm) to 11.4 MPa (25 mm), consistent with a bending-dominated deformation mode.
- The energy absorption density (EAD) increases with strain ranging from 0.67 J/cm^3^ (4.6 mm) to 0.03 J/cm^3^ (25 mm) at 30% strain, with thinner-core panels (P3 and P4) showing greater efficiency per unit volume, especially beyond 30–40% strain.

Among the tested configurations, the intermediate-height core (P2) exhibited the best overall balance among stiffness, load-bearing capacity, and energy dissipation. It should be noted that, in the investigated configurations, the highest load-bearing capacity in terms of peak stress is achieved by thinner cores, whereas thicker cores mainly promote increased deformability and progressive collapse. These findings are consistent with previous literature on corrugated and lattice-core structures and suggest that the trapezoidal corrugated design may be a promising solution for lightweight applications requiring efficient energy absorption, such as in the transportation or impact protection sectors.

## Figures and Tables

**Table 1:** Geometric characteristics of the tested sandwich panels. The table reports core height, face sheet thickness, core wall thickness, and panel area.

Sandwich Panel	Height (mm)	Face Sheet Thickness (mm)	Core Height (mm)	Core Wall Thickness (mm)	Panel Area (mm^2^)
P1	25.0	1	23	0.3	8008
P2	10.4	1	8	0.3	7480
P3	6.2	1	4	0.3	7225
P4	4.6	1	2	0.3	7395

**Table 2:** Estimated values of peak stress (σ_max_), strain at peak, initial stiffness, and plateau stress for the four trapezoidal-core sandwich panels with varying thickness.

Sample	ε_el_ (%)	σ_el_ (MPa)	Strain at Peak (%)	Peak Stress (MPa)	Estimated Stiffness (MPa)	σ_plateau_ (MPa)
P1	3.5	0.4	3.9	0.5	11.4	0.21
P2	11.5	1.5	12.6	1.6	13.0	0.45
P3	15.0	2.6	17.2	3.2	17.3	-
P4	21.0	3.9	35.8	6.2	18.6	-

**Table 3:** Energy absorption density (EAD) values obtained for each sandwich panel type (P1–P4) at 30% and 50% strain, with three repeated tests per configuration.

	EAD_ε=30%_ (J/cm^3^)				EAD_ε=50%_ (J/cm^3^)			
	P1	P2	P3	P4	P1	P2	P3	P4
Test 1	0.034	0.16	0.40	0.50	0.068	0.30	1.02	-
Test 2	0.029	0.20	0.47	0.89	0.037	0.42	1.21	-
Test 3	0.030	0.20	0.45	0.62	0.037	0.45	1.11	-

**Table 4:** Mean and standard deviation of energy absorbed density (EAD) at 30% and 50% strain for each panel configuration. * P4 does not have valid values at 50% strain, therefore it is not possible to calculate the mean and standard deviation for that deformation level.

Panel	EAD_ε=30%_ (J/cm^3^)	EAD_ε=50%_ (J/cm^3^)
P1	0.031 ± 0.003	0.047 ± 0.018
P2	0.187 ± 0.023	0.390 ± 0.076
P3	0.440 ± 0.029	1.113 ± 0.095
P4	0.670 ± 0.197	- *

## Data Availability

The original contributions presented in this study are included in the article. Further inquiries can be directed to the corresponding author.
